# Implementation of the acutely presenting older patient (APOP) screening program in routine emergency department care: A before-after study

**DOI:** 10.1007/s00391-020-01837-9

**Published:** 2021-01-20

**Authors:** Laura C. Blomaard, Bas de Groot, Jacinta A. Lucke, Jelle de Gelder, Anja M. Booijen, Jacobijn Gussekloo, Simon P. Mooijaart

**Affiliations:** 1 Department of Internal Medicine, section Geriatrics, Leiden University Medical Center, 9600, 2300 RC Leiden, The Netherlands; 2 Department of Emergency Medicine, Leiden University Medical Center, Leiden, The Netherlands; 3 Department of Emergency Medicine, Spaarne Gasthuis, Haarlem, The Netherlands; 4 Department of Public Health and Primary Care, Leiden University Medical Center, Leiden, The Netherlands; 5 Institute of Evidence-Based Medicine in Old Age | IEMO, Leiden, The Netherlands

**Keywords:** Frail elderly, Geriatric assessment, Geriatric emergency medicine, Implementation science, Quality improvement, Gebrechliche ältere Menschen, Geriatrische Beurteilung, Geriatrische Notfallmedizin, Implementierungswissenschaft, Qualitätsverbesserung

## Abstract

**Objective:**

The aim of this study was to evaluate the effects of implementation of the acutely presenting older patient (APOP) screening program for older patients in routine emergency department (ED) care shortly after implementation.

**Methods:**

We conducted an implementation study with before-after design, using the plan-do-study-act (PDSA) model for quality improvement, in the ED of a Dutch academic hospital. All consecutive patients ≥ 70 years during 2 months before and after implementation were included. The APOP program comprises screening for risk of functional decline, mortality and cognitive impairment, targeted interventions for high-risk patients and education of professionals. Outcome measures were compliance with interventions and impact on ED process, length of stay (LOS) and hospital admission rate.

**Results:**

Two comparable groups of patients (median age 77 years) were included before (*n* = 920) and after (*n* = 953) implementation. After implementation 560 (59%) patients were screened of which 190 (34%) were high-risk patients. Some of the program interventions for high-risk patients in the ED were adhered to, some were not. More hospitalized patients received comprehensive geriatric assessment (CGA) after implementation (21% before vs. 31% after; *p* = 0.002). In 89% of high-risk patients who were discharged to home, telephone follow-up was initiated. Implementation did not influence median ED LOS (202 min before vs. 196 min after; *p* = 0.152) or hospital admission rate (40% before vs. 39% after; *p* = 0.410).

**Conclusion:**

Implementation of the APOP screening program in routine ED care did not negatively impact the ED process and resulted in an increase of CGA and telephone follow-up in older patients. Future studies should investigate whether sustainable changes in management and patient outcomes occur after more PDSA cycles.

**Supplementary Information:**

The online version of this article (10.1007/s00391-020-01837-9) contains supplementary material, which is available to authorized users.

## Abstract (de)

**Zielsetzung:**

Beurteilung der Auswirkungen der Implementierung des Screening-Programms für ältere Patienten (APOP) in der Routineversorgung der Notaufnahme (ED) kurz nach der Anwendung.

**Methoden:**

Eine Implementierungsstudie mit Vorher-Nachher-Design wurde unter Verwendung des Plan-do-study-act(PDSA)-Modells zur Qualitätsverbesserung in der Notaufnahme eines niederländischen akademischen Krankenhauses durchgeführt. Alle konsekutiven Patienten ≥70 Jahre während der 2 Monate vor und nach der Implementierung wurden eingeschlossen. Das APOP-Screening-Programm umfasst eine Untersuchung auf das Risiko von Funktionseinschränkungen, Mortalität und kognitiver Beeinträchtigung, gezielte Interventionen für Hochrisikopatienten und die Schulung von Fachpersonal. Die Ergebnisse wurden anhand der Compliance mit den Interventionen und den Auswirkungen auf die Abläufe in der Notaufnahme, die Verweildauer (LOS) und die Krankenhauseinweisungsrate gemessen.

**Ergebnisse:**

Zwei vergleichbare Gruppen von Patienten (mittleres Alter: 77 Jahre) wurden vor (*n* = 920) und nach (*n* = 953) der Implementierung eingeschlossen. Nach der Implementierung wurden 560 (59 %) Patienten gescreent, von denen 190 (34 %) Hochrisikopatienten waren. Die Interventionen für Hochrisikopatienten in der Notaufnahme wurden teilweise eingehalten. Mehr hospitalisierte Patienten erhielten nach der Implementierung ein umfassendes geriatrisches Assessment (CGA; 21 % vorher vs. 31 % nachher; *p* = 0,002). Bei 89 % der Hochrisikopatienten, die nach Hause entlassen wurden, wurde eine telefonische Nachbetreuung eingeleitet. Die Implementierung hatte keinen Einfluss auf die mediane Verweildauer in der Notaufnahme (202 min vorher vs. 196 min nachher; *p* = 0,152) oder die Krankenhauseinweisungsrate (40 % vorher vs. 39 % nachher; *p* = 0,410).

**Schlussfolgerung:**

Die Implementierung des APOP-Screening-Programms in die routinemäßige Versorgung in der Notaufnahme hatte keine negativen Auswirkungen auf den ED-Prozess und führte zu einer Zunahme von CGA und telefonischer Nachsorge bei älteren Patienten. In zukünftigen Studien sollte untersucht werden, ob nach weiteren PDSA-Zyklen nachhaltige Veränderungen im Management und in den Patientenergebnissen auftreten.

## Introduction

Older patients form an increasing proportion of emergency department (ED) admissions worldwide and are at higher risk of adverse health outcomes compared to younger patients [1]. The presence of multiple comorbidities, cognitive disorders and atypical disease presentations requires more staff time and resources [2], increases ED length of stay (LOS) and poses organizational challenges [3, 4]. A comprehensive geriatric assessment (CGA) is an effective method to improve older patients’ outcomes [5] but CGA is time-consuming and therefore cannot be routinely applied to every older patient attending the ED. Alternatively, a two-step approach can be used with identification of patients with the highest risk of adverse outcome as a first step, followed by targeted interventions according to the principles of CGA [6, 7]. To this end, several screening instruments and interventions have been specifically developed for older patients in the ED [8, 9] yet few have successfully been disseminated in clinical ED practice.

The acutely presenting older patient (APOP) screening program consists of screening with the APOP screener followed by interventions aimed to improve overall ED care and follow-up of older patients [10]. The program was implemented in routine ED care in the Leiden University Medical Center (LUMC) together with an education program to enhance awareness amongst nurses and doctors working in the ED. There is extensive evidence that effective implementation of complex interventions can be associated with better outcomes in various settings outside the ED, which implicates that evaluation of implementation is an absolute necessity in program evaluation [11, 12]. One of the important reasons why screening of older ED patients is rarely carried out in routine care, is the fact that little is known about the practical issues and feasibility of implementation in everyday ED practice [13], although it was recently shown that administration of the APOP screener is feasible in routine ED practice [14].

In the present study we aimed to evaluate the effects of implementation of the APOP screening program in routine ED care by assessing the compliance with interventions in the ED, during hospital admission and after discharge, and the impact on process of care measures, shortly after implementation. We hypothesized that the implementation of the screening program would not negatively influence the usual ED process, for example no prolongation of the ED stay and it would result in improvement of the care for older patients, for example the increase in geriatric assessments.

## Methods

### Study design

This was a prospective study investigating the effects of implementation of the APOP screening program with a before-after design, conducted in the ED of the LUMC. The APOP program was kicked-off as part of routine ED care on 1 March 2018. Data were collected during a 2-month observation period before implementation (“before”) from 4 December 2017 until 2 February 2018, and during 2 months after implementation (“after”) from 2 April 2018 until 3 June 2018. All consecutive patients aged 70 years and older attending the ED during these periods were included in the study. The medical ethics committee of the hospital waived the necessity for formal approval of this study as it closely follows routine care. All patient data were anonymized before analyses were executed. The standards for reporting implementation studies (StaRI) were used to present the study [15].

### Context

The APOP screening program was implemented in the context of an ageing Dutch population where the financial crisis forced governments to stimulate older patients to stay at home longer, while the capacity of home care and nursing homes decreased seriously in the last years. The Netherlands has ~38,000 hospital beds, ~115,000 nursing home beds and ~13,000 general practitioners available for a population of 17 million people. The increased number of older patients presenting to the ED has been a constant debate in politics, and older patients are believed to be the cause of increasing overcrowding of Dutch EDs. This resulted in more attention for older ED patients and an upcoming motivation of ED care providers to improve care for this population.

### Setting

The LUMC is a tertiary care centre with ~26,000 ED visits per year, of which approximately 20% are patients aged ≥ 70 years. In the ED, a triage nurse prioritizes patients based on the disease severity, using the Manchester triage system (MTS) [16]. Patients who bypass ED triage are patients eligible for thrombolytic treatment and patients with an indication for telemetry or cardiac catheterization who are admitted to the emergency cardiac care unit. The ED is staffed each day of the week for 24 h by ED nurses, ED physicians, ED residents and residents of other specialties. When hospitalization is indicated after ED treatment, most patients are admitted to the acute medical unit (AMU), which is a 24-bed unit for admission up to 48 h of medical, surgical and selected neurological patients.

### Implementation strategy

**Fig. 1:**
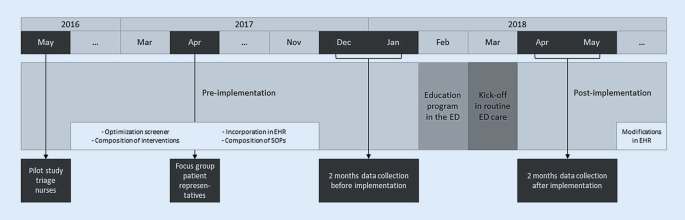
Overview of the implementation process and data collection periods. Data were collected in multiple periods during the implementation process. In the present study, we evaluated data collected from patients aged ≥ 70 years visiting the emergency department (ED) during the 2‑month observation periods “before” and “after” implementation of the APOP screening program. *EHR* electronic health record, *SOP* standard operating procedures

The implementation strategy was guided by the plan-do-study-act (PDSA) model for quality improvement [17, 18]. In the preimplementation phase, we used recurring PDSA cycles and assessed barriers and facilitators of the program from pilot studies with ED nurses and focus groups with patient representatives (Fig. 1). The received input was taken into account during the optimization of the APOP screener [10] and the facilitation of the program in the electronic health records (EHR) and standard operating procedures (SOP). We carried out an education program for ED personnel to enhance awareness during 1 month before the kick-off in routine care. A complete description of the implementation strategy and the education program [19] can be found in appendix 1.

### Outline of the APOP screening program

**Fig. 2:**
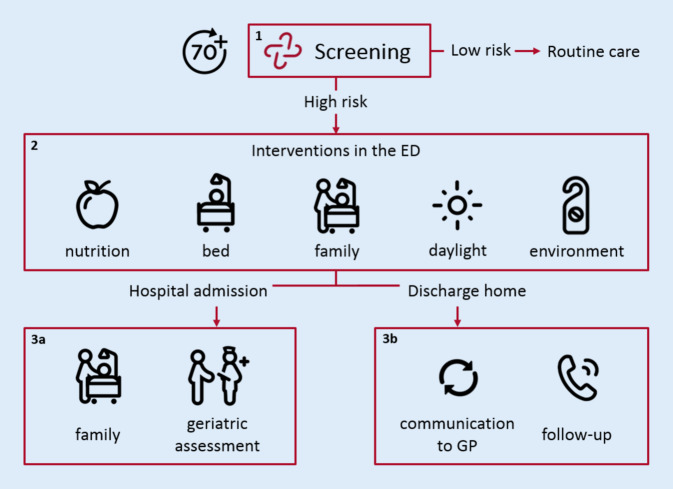
Overview of the acutely presenting older patient (APOP) screening program. The APOP screening program consists of three parts: firstly, screening older patients for risk of functional decline/mortality and signs of impaired cognition, secondly targeted interventions for high-risk patients in the emergency department (ED) and thirdly interventions for high-risk patients who are hospitalized or discharged home

The APOP screening program was developed for ED patients aged ≥ 70 years and consists of three parts (Fig. 2):

#### 1. Screening.

The APOP screener can be administered in 90 s and identifies the patients’ individual risk of 90-day functional decline and/or mortality and signs of impaired cognition in the ED [10]. All patients aged ≥ 70 years are eligible for screening after routine ED triage. In this study we excluded patients who bypassed triage and patients who were triaged to the immediate urgency level (MTS category “red”), because the APOP screener was not developed and validated for this population. Screening results are saved in the EHR and are visible for all care providers. Patients with a low risk according to screening receive routine care. Patients are at high risk when having a 45% or higher risk of functional decline and/or mortality within 90 days or when having signs of impaired cognition [10, 14].

#### 2. Interventions for high-risk patients in the ED.

A high risk leads to follow-up actions and interventions. Interventions were based on recommendations from geriatric emergency medicine guidelines [6, 20] and were adjusted for use in the Dutch ED setting (appendix 1). The APOP program is a broader program, but in this study we describe the interventions which were evaluated. A full description of these interventions is shown in appendix 2. Physicians and nurses are advised to execute interventions in the ED to increase comfort, family involvement and delirium prevention.

#### 3a Interventions for high-risk patients admitted to the hospital.

Interventions can be conducted in an early phase when high-risk patients are hospitalized. Care providers are advised to avoid a prolonged ED LOS and to arrange family involvement during transfer to the ward. The geriatric consulting team is informed automatically by the EHR to arrange a comprehensive geriatric assessment (CGA) during hospital admission.

#### 3b Interventions for high-risk patients discharged home from the ED.

The GP is informed about the high-risk result automatically by the EHR in the discharge letter from ED physicians. For high-risk patients who are discharged home from the ED, telephone follow-up is initiated within 24 h after discharge. The ED nurses contact patients to find out if they have remaining questions about the ED treatment and if they need any help (i.e. clarification of instructions).

### Outcomes

The present study had the following outcome measures: Firstly, compliance with interventions of executed interventions in the ED, during hospital admission or after discharge. Secondly, impact on process of ED care measures: ED LOS and hospital admission rate.

### Data collection

#### Patient characteristics and organizational factors

In order to evaluate potential differences between the two data collection periods, we collected patient characteristics and organizational factors before and after implementation. Patient characteristics were collected from the EHR on demographics (age, gender) and severity of disease (Charlson comorbidity index, CCI [21], arrival by ambulance, MTS triage urgency and chief complaint [16] and the specialist first assigned to treat the patient in the ED). To measure organizational factors on a patient level, we used real-time observations in the ED. During the “before” and “after” data collection periods medical students were present in the ED 7 days per week (8.00a.m.–11.00p.m.). Observed organizational factors were: the total number of ED patients at arrival day, the actual number of ED patients at arrival time, the number of occupied AMU beds at arrival time and the national emergency department overcrowding score (NEDOCS) at arrival and departure times [22]. Our hospital uses an adapted, but not yet validated, NEDOCS applicable for Dutch EDs (NEDOCS 0–50 = normal, 51–100 busy, 101–140 overcrowded, 141–180 severe, > 181 disaster).

##### 1. Screening rate.

After implementation, data were collected on the number of patients with executed APOP screening and the results of screening. The number of screened patients divided by the total number of older patients per day yielded the screening rate [14].

##### 2. Compliance with interventions—in the ED.

The compliance with interventions was measured by absolute numbers of executed interventions in real-time observed older patients “before” and “after” implementation. Additionally, we evaluated the compliance in high-risk patients after implementation. Observations of executed interventions were done from a central place in the ED where most treatment rooms were visible. During the whole ED visit we observed whether older patients: 1) were offered nutrition, 2) were placed in a bed instead of a gurney, 3) had family present and 4) were placed in a room with daylight. The stressfulness of the ED environment was measured by the number of involved care providers, the number of treatment room door movements and the proportion of time the treatment room door was open for whole ED LOS. The ED personnel were not informed about the reason for observation.

##### 3a Compliance with interventions—hospital admission.

For older patients hospitalized in our hospital wards, we observed real time the accompaniment by family when leaving the ED. Consultation of the geriatric team for CGA during admission was collected from the EHR. The compliance was quantified by the number of patients who received CGA divided by the total number of hospitalized older patients.

##### 3b Compliance with interventions—discharge home.

The novel interventions communication to GP and telephone follow-up were collected after implementation from the EHR. The compliance of communication to GP was quantified by the number of high-risk patients with an automatically incorporated discharge letter divided by the total number of high-risk discharged patients. Telephone follow-up compliance was quantified by the number of high-risk patients who received follow-up divided by the total number of high-risk patients discharged home.

#### Impact on process of ED care

Process of care measures were collected from the EHR and were available for all triaged older ED patients before and after implementation. The ED LOS was measured by subtraction of the ED arrival time from the departure time. Hospital admission rate was measured by the number of patients hospitalized from the ED divided by the total number of older ED patients, during the before and after observation period.

#### Sample size calculation

The sample size was calculated on ED LOS and hospital admission rate. In a previous analysis of our ED, older patients had a median ED LOS of 189 min (interquartile range, IQR, 125–264 min) and the hospital admission rate was 43% [23]. We considered a change of 15 min ED LOS and 7% hospital admission rate as relevant. To detect a difference for the groups before and after with 80% power and 5% significance level, per group 891 patients were needed for ED LOS and 796 patients for hospital admission rate.

### Statistical analyses

Continuous data are presented as mean (standard deviation, SD) if normally distributed, and as median (IQR) if skewed. Categorical data are presented as numbers and percentages (*n*, %). The following statistical tests were used to assess differences in patient characteristics, organizational factors and compliance with interventions between the after and before period: independent samples t‑test for normally distributed data, Mann-Whitney U-test for skewed data and χ^2^-test for categorical data.

To analyze the impact on process of ED care measures univariable logistic regression was performed, with ED LOS (<240 min, ≥ 240 min) and hospital admission (yes, no) as dependent variables and the inclusion period “after” vs. “before” as the independent variable of interest. With multivariable logistic regression we adjusted for age and gender (model 1) and for age, gender and all significantly different variables between the “after” and “before” period (model 2). The results are presented as odds ratios (OR) with 95% confidence intervals (95% CI). A *p*-value < 0.05 was determined as statistically significant. Statistical analyses were performed using IBM SPSS Statistics version 25 (IBM Corp., Armonk, NY, USA).

## Results

**Fig. 3:**
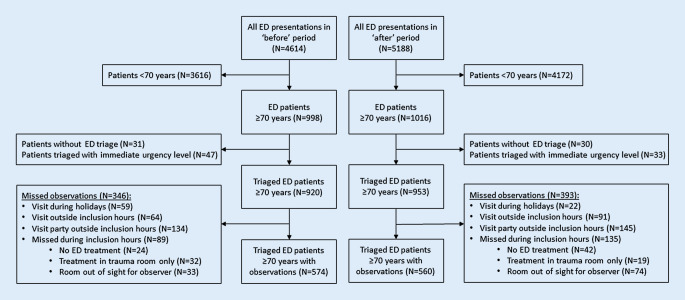
Flowchart of study population. All consecutive patients aged ≥ 70 years visiting the emergency department (ED) during the 2‑month observation periods “before” and “after” implementation of the APOP screening program were included, except for patients who bypassed ED triage or patients who were triaged to the immediate urgency level. The screening rate was measured in triaged ED patients ≥ 70 years in the “after” period. Compliance with interventions was compared in the “before” and “after” period, using real-time observations of ED patients ≥ 70 years. Process of care measures were compared between all triaged ED patients ≥ 70 years in the “before” and “after” period

During the 2‑month observation period before implementation (“before”) 4614 patients visited the ED of which 920 (20%) were patients aged ≥ 70 years who were triaged at ED arrival. In the 2‑month observation period after implementation (“after”) 953 out of 5188 (18%) ED patients were triaged patients aged ≥ 70 years. Of all triaged older patients, 62% (*N* = 574) was observed “before” and 59% (*N* = 560) “after” in order to evaluate the compliance with interventions (Fig. 3).

### Patient characteristics and organizational factors

Table 1 shows the characteristics and organizational factors on a patient level “before” and “after”. The median age of patients was the same in both periods: 77 years (IQR 73–82 years). Severity of disease indicators were comparable “before” and “after”. Organizational factors “before” and “after” differed: the mean total number of ED patients per day was higher in the “after” period (77 patients (SD 10) before vs. 83 patients (SD 12) after; *p* < 0.001), but the median NEDOCS at time of ED departure was lower “after” (62 (IQR 42–80) before vs. 57 (IQR 38–72) after; *p* = 0.001).

**Table 1:** Patient characteristics and organizational factors before and after implementation

	Before (*N* = 920)	After (*N* = 953)	*p*-value
*Demographics*			
Age, years median (IQR)	77 (73–82)	77 (73–82)	0.372
Male, *n* (%)	439 (47.7)	471 (49.4)	0.460
*Severity of disease indicators*			
Charlson comorbidity index, median (IQR)	5 (4–6)	5 (4–7)	0.014
Arrival by ambulance, *n* (%)	316 (34.3)	293 (30.7)	0.096
*Triage urgency, n (%)*			0.585
>1 h (green and blue)	206 (22.4)	219 (23.0)	
<1 h (yellow)	449 (48.8)	443 (46.5)	
<15 min (orange)	265 (28.8)	291 (30.5)	
*Chief complaint, n (%)*			0.533
Minor trauma	256 (28.0)	276 (29.3)	
Malaise	237 (25.9)	247 (26.2)	
Dyspnea	121 (13.2)	96 (10.2)	
Abdominal pain	97 (10.6)	91 (9.7)	
Chest pain	61 (6.7)	75 (8.0)	
Loss of consciousness	44 (4.8)	41 (4.4)	
Major trauma	13 (1.4)	15 (1.6)	
Mental health problems	6 (0.7)	10 (1.1)	
Other	80 (8.7)	91 (9.7)	
*First assigned specialist in ED, n (%)*			<0.001
ED physician	400 (44.3)	381 (42.1)	
Internal medicine	147 (16.3)	82 (9.1)	
Neurology	104 (11.5)	104 (11.5)	
Surgery	63 (7.0)	54 (6.0)	
Cardiology	59 (6.5)	71 (7.8)	
Other	129 (14.3)	214 (23.6)	
*Observed organizational factors on patient level*			
Total number of ED patients on arrival day, mean (SD)	77 (10)	83 (12)	<0.001
Number of ED patients at time of arrival, mean (SD)	13 (5)	13 (5)	0.170
Number of occupied AMU beds at time of arrival, mean (SD)	18 (4)	17 (4)	0.002
NEDOCS at time of starting medical treatment, median (IQR)	50 (27–70)	51 (28–68)	0.998
NEDOCS at time of departure from ED, median (IQR)	62 (42–80)	57 (38–72)	0.001

Demographics and severity of disease indicators were collected from electronic health records. Organizational factors were collected by real-time observations during the ED visit

Missing data

Before: 36 CCI, 5 chief complaint, 18 first assigned specialist, 4 number of ED patients at time of arrival, 4 number of occupied AMU beds, 56 NEDOCS at time of start treatment, 57 NEDOCS at time of departure

After: 56 CCI, 11 chief complaint, 47 first assigned specialist, 1 number of ED patients at time of arrival, 2 number of occupied AMU beds, 75 NEDOCS at time of start treatment, 38 NEDOCS at time of departure

*N* number, *IQR* interquartile range, *SD* standard deviation, *AMU* acute medical unit, *NEDOCS* national emergency department overcrowding score, *ED* emergency department

#### 1. Screening rate.

During the 2‑month observation period “after” implementation 560 (59%) of the 953 older patients were screened [14]. As a result of screening, 190 (34%) patients were classified as having a high risk, which made them eligible for interventions.

#### 2. Compliance with interventions—in the ED.

Compliance with interventions was evaluated by comparison of executed interventions between all real-time observed older patients “before” and “after” (Table 2). In the “after” period older patients more often received nutrition in the ED (7% before vs. 12% after; *p* = 0.004). No improvements were found in nursing on a bed (35% before vs. 27% after; *p* = 0.004), family presence (89% before vs. 84% after; *p* = 0.043) and room with daylight (30% before vs. 34% after; *p* = 0.235). Proxies for stressfulness of the ED environment were better “after” for median number of door movements (40, IQR 24–62 before vs. 25, IQR 15–40 after; *p* < 0.001) and median number of involved staff (7, IQR 5–10 before vs. 5, IQR 4–7 after; *p* < 0.001).

**Table 2:** Compliance with interventions before vs. after implementation and compliance with interventions for high-risk screened patients after implementation

	Total group of observed older patients					High-risk screened observed patients	
	Before		After		*p*-value	After	
	Number observed	Compliance	Number observed	Compliance		Number observed	Compliance
*Interventions in the ED*							
Received nutrition, *n* (%)	540	37 (6.9)	528	63 (11.9)	0.004	111	27 (24.3)
Nursed on bed, *n* (%)	542	190 (35.1)	534	144 (27.0)	0.004	114	42 (36.8)
Family present, *n* (%)	536	475 (88.6)	518	437 (84.4)	0.043	113	98 (86.7)
Room with daylight, *n* (%)	523	158 (30.2)	508	171 (33.7)	0.235	108	44 (40.7)
Number of door movements, median (IQR)	523	40 (24–62)	513	25 (15–40)	<0.001	111	31 (17–46)
Number of staff involved, median (IQR)	524	7 (5–10)	513	5 (4–7)	<0.001	111	6 (4–8)
Proportion open door time (%)^a^, median (IQR)	423	15 (5–31)	508	16 (6–33)	0.190	110	22 (7–38)
*Interventions at hospital admission*^b^							
Family present during admission, *n* (%)	216	174 (80.6)	174	147 (84.5)	0.312	46	37 (80.4)
Geriatric assessment, *n* (%)	343	72 (21.0)	365	114 (31.2)	0.002	91	65 (71.4)
*Interventions at discharge home*^c^							
Communication to GP, *n* (%)	NA	NA	NA	NA	NA	80	57 (71.3)
Telephone follow-up, *n* (%)	NA	NA	NA	NA	NA	79	70 (88.6)

Total number of triaged patients ≥ 70 years before *N* = 920; after *N* = 953. Patients were observed real-time when visiting the ED between 8 a.m. and 11 p.m. Total number of observed triaged patients ≥ 70 years before *N* = 574; after *N* = 560. Total number of high-risk screened patients after implementation *N* = 190

*N* number, *IQR* interquartile range, *NA* not applicable, *GP* general practitioner, *ED* emergency department

^a^Proportion of time the treatment room door was open for whole ED length of stay in percentage

^b^Numbers of admitted patients in our hospital: before *N* = 362, after *N* = 368, high-risk screened patients *N* = 92

^c^Numbers of patients discharged home: before *N* = 467, after *N* = 488, high-risk screened patients *N* = 80

#### 3a. Compliance with interventions—hospital admission.

In total 362 (40%) patients “before” and 368 (39%) patients “after” were admitted to the hospital. More hospitalized patients received CGA during admission “after” compared to “before” (21% before vs. 31% after; *p* = 0.002). Of a total of 92 admitted high-risk patients after implementation 65 (71%) patients received CGA.

#### 3b. Compliance with interventions—discharge home.

After implementation 80 high risk patients were discharged home. In 57 (71%) patients, the high-risk result was communicated to the GP. Telephone follow-up was initiated in 70 (89%) patients. In total 81% of patients were reached by telephone, of whom 37% required clarification of home care instructions.

#### Impact on process of ED care

In Table 3, process of ED care outcomes are compared for all included patients “before” and “after”. The median ED LOS was comparable between both groups with 202 min (IQR 133–290min) before vs. 196 min (IQR 133–265min) after; *p* = 0.152. No prolonged ED LOS in the “after” period was found, after adjusting for possible confounders (OR 0.88, 95%CI 0.66–1.17, *p* = 0.371) (supplemental Table 1). Hospital admission rates were comparable between both groups: 362 (40%) patients before vs. 368 (39%) patients after; *p* = 0.642. After adjustment for possible confounders, the hospital admission rate in the “after” period was lower (OR 0.68, 95%CI 0.50–0.92, *p* = 0.013).

**Table 3:** Process of ED care outcomes for patients before and after implementation

	Before (*N* = 920)	After (*N* = 953)	*p*-value
ED LOS (min), median (IQR)	202 (133–290)	196 (133–265)	0.152
Hospital admission after ED visit, *n* (%)	362 (40.0)	368 (38.9)	0.642

Missing data

Before: 2 ED LOS, 15 disposition after ED visit. After: 2 ED LOS, 8 disposition after ED visit

*N* number, *IQR* interquartile range, *LOS* length of stay, *ED* emergency department

## Discussion

In this study, the first effects of implementation of the APOP screening program in routine ED care were evaluated after 1 month by assessing the compliance with interventions and the impact on process of care measures. Interventions for high-risk patients in the ED were partly adhered to. Implementation of the program resulted in increased numbers of executed CGAs during hospitalization, communication of screening results to the GP and telephone follow-up after ED discharge. Implementation had no major effects on ED LOS and hospital admission.

To the best of our knowledge this is the first study evaluating the implementation of a multicomponent screening program for older patients comprising screening and targeted interventions in routine ED care. In a recent substudy we showed that implementation of the APOP screener was feasible with a screening rate of 59% [14]. Compared to other studies [13, 24, 25] our screening rate assessed shortly after implementation in routine ED care is relatively high. A screening rate of 100% is difficult to achieve because the time restraints inherent to a busy ED will prevent nurses administering the screener. Since there are only few ED multicomponent studies published [26] we are only able to compare single components. In one study, telephone follow-up for all older ED patients resulted in 97% successfully contacted patients of which 40% required clarification of home care instructions [27], comparable to our results in high-risk older patients. The use of a clinical risk prediction tool to select high-risk patients and target interventions to those patients most likely to benefit, the increased proportion of patients who receive CGA and the improved communication of screening results to the GP have been associated with improved patient outcomes in other settings [8, 9, 28]. Definitive proof of (cost)effectiveness of the APOP screening program on patient outcomes, such as functional decline, should come from future studies, for example by using a multicenter stepped-wedge design [29].

The present study has several important findings for clinical practice. Firstly, implementation of screening in the ED resulted in improved execution of some individual interventions for older patients during their ED stay, i.e. adequate nutrition. However, the intervention “presence of family” did not increase, probably because this was already very high before implementation, i.e. a ceiling effect. The interventions “nursed on bed” and “room with daylight” also did not improve, probably because they were less feasible due to a lack of capacity (in our ED there are few beds and rooms with daylight available). Secondly, program implementation resulted in a significant increase in the number of executed CGAs, which has been shown to be an effective method to improve outcomes [5]. In 71% of the high-risk patients CGAs were executed during hospitalization. Therefore, although interventions in the ED are not always executed, screening is a useful first step to ensure that high-risk patients receive optimal care during hospitalization. The same holds for high-risk patients discharged home from the ED, of which 79% were reached for telephone follow-up. Finally, implementation of our screening program did not lead to prolonged ED LOS or more hospital admissions. After adjustment for the small differences in the before and after group, there even seem to be less hospital admissions after which is important because impact on capacity is relevant to the feasibility and sustainability of the program.

The repetitive use of the PDSA model as a framework for our implementation strategy helped in understanding barriers and facilitators of implementation [14]. Continuation of future PDSA cycles can help to further improve compliance in our ED and can also help others to start implementation of this screening program elsewhere. The results of the present study are therefore the starting point for new evaluation cycles of the program. Until now, we mainly focused our implementation strategy on the ED nurses, the executors of the screening, which also resulted in mainly nurse-led interventions for high-risk patients. In future, we aim to focus more on physicians and use additional education to increase their awareness and promote a more holistic clinical assessment of older ED patients. Moreover, the interventions of our program were based on recommendations from international guidelines and quality indicators [6, 20] and could be updated according to recent recommendations [30]. If other EDs would like to implement a screening program for older patients they can learn from our limitations and adjust their expectations accordingly, i.e. ensure the presence of rooms with daylight and focus on adequate nutrition during an ED stay.

Our study has several strengths. Firstly, to the best of our knowledge this is the first implementation study evaluating screening and interventions for older patients in routine ED care on a large scale, using real-time observations. Secondly, our implementation strategy was guided by the generally used PDSA model for quality improvement, resulting in good understanding of barriers and facilitators of implementation. Lastly, the screening program was implemented and evaluated in an unselected population of older ED patients, which is therefore generalizable to other ED populations.

Our study also has several limitations. Firstly, the before-after study design has time and seasonal variation as a limitation; however, there were no contextual changes between the two data collection periods. Also, we could not detect substantial differences in patient characteristics between the “before” and “after” group. Furthermore, the main outcome measures for the evaluation of the program were process measures—the proportion of hospitalized patients with geriatric assessment and the proportion of discharged patients with follow-up telephone calls—which are likely unaffected by time period or seasonal variation. Secondly, before implementation older patients could not be screened. Therefore, we could only compare compliance with interventions on the level of total group ED patients ≥ 70 years in the before and after periods. Small improvements in compliance with interventions in high-risk patients might therefore have been missed. Finally, the program was implemented in one tertiary care center which limits generalizability.

In conclusion, implementation of the APOP screening program in routine ED care did not negatively impact the ED process and resulted in an increase of CGA and telephone follow-up in older patients. Since this was a first evaluation shortly after implementation, future studies should investigate whether sustainable changes in management and patient outcomes occur after more PDSA cycles.

## Supplementary Information

Supplemental table 1. Risk of prolonged ED LOS and hospital admission after implementation compared to before
Appendix 1. Implementation strategy
Appendix 2. Overview of the APOP screening program advices for interventions
Standards for Reporting Implementation Studies: the StaRI checklist
