# Supplementary material for: Implementation of the acutely presenting older patient (APOP) screening program in routine emergency department care: A before-after study
Source: Z Gerontol Geriatr. 2021 Jan 20;54(2):113–21. doi: 10.1007/s00391-020-01837-9 (PMC7946672; doi:10.1007/s00391-020-01837-9)
Supplement: Supplementary file 1 — Supplemental table 1. Risk of prolonged ED LOS and hospital admission after implementation compared to before Appendix 1. Implementation strategy Appendix 2. Overview of the APOP screening program advices for interventions Standards for Reporting Implementation Studies: the StaRI checklist [file 391_2020_1837_MOESM1_ESM.docx]

**SUPPLEMENTARY FILES**

| **Supplemental table 1.** Risk of prolonged ED LOS and hospital admission after implementation compared to before (reference) | | |
| --- | --- | --- |
|  | **OR (95% CI)** | **p-value** |
| **ED LOS ≥240 min. ‘After’ vs ‘Before’** |  |  |
| crude | 0.86 (0.71-1.04) | 0.126 |
| model 1 | 0.86 (0.71-1.05) | 0.134 |
| model 2 | 0.88 (0.66-1.17) | 0.371 |
| **Hospital admission ‘After’ vs ‘Before’** |  |  |
| crude | 0.96 (0.79-1.15) | 0.642 |
| model 1 | 0.95 (0.79-1.15) | 0.621 |
| model 2 | 0.68 (0.50-0.92) | 0.013 |
| OR = Odds ratio; CI = confidence interval; LOS = length of stay  Risk of prolonged ED LOS and hospital admission for patients included after implementation of the APOP screening program (N=953) compared to patients included before implementation (N=920).  Model 1: adjusted for age, gender  Model 2: adjusted for age, gender, CCI, first assigned specialist, total number of ED patients on arrival day, number of occupied AMU-beds at time of arrival and NEDOCS at departure time | | |

**Appendix 1.** Implementation strategy

**Pre-implementation phase**

Implementation planning for this study began with formal approval of the division boards of our hospital after the construction of a multidisciplinary project-team consisting of an ED physician, resident ED physician, ED-nurse, internist-geriatrician, geriatric nurse, AMU nurse, researchers and a general practitioner. Based on project-team experiences and literature the implementation strategy, outline of interventions and education program was developed. For the outline of the interventions we used usable elements of Comprehensive Geriatric Assessment (CGA), taking into account the recommendations from international guidelines and quality indicators.(6, 20) There is no evidence yet whether these interventions improve outcomes for older patients. From the recommendations in international literature we selected interventions which were practicable to implement in routine care in the Dutch ED setting. In addition, we also selected interventions based on project-team experience and input from focus groups with patient representatives and general practitioners.

Implementation strategy

Our implementation strategy was guided by the plan-do-study-act (PDSA) model for quality improvement.(17) In the first PDSA cycle the use of the screening instrument in practice was evaluated in a pilot study with ED triage-nurses. We assessed readiness to adopt the interventions, specific uptake goals and barriers and facilitators. The received input was taken into account during the development of the final screening instrument and the facilitation of the program.(10) For example, we excluded a question about polypharmacy in the final screening instrument because it took too much time to execute in practice. Triage-nurses experienced a barrier to ask for dementia, one of the questions in the APOP screener. We therefore collected input from patient representatives on how this question could best be asked. Data was collected with focus group sessions with the older patient council of our hospital (Ouderenberaad Zorg en Welzijn Zuid-Holland Noord). Their input was written down in the standard operating procedures of the APOP screening program. The most important facilitator for use of the screening instrument in routine care turned out to be implementation in the Electronic Health Records (EHR). This result was the starting point for following PDSA cycles in which the screening instrument, signals of high risk results and automatic orders were incorporated in the EHR.

Education program

Education was used to enhance awareness and increase knowledge of the ED team of different care needs of older people, especially aspects relating to frailty and geriatric syndromes, for which a broader, more holistic intervention is considered to be best practice. The other rationale for education was to influence adoption of the screening program by clarification of all program components. The education program was developed during the pre-implementation phase by the members of the multidisciplinary project-team. Outline for the education program was based on recommendations from the Curriculum for Geriatric Emergency Medicine designed by the European Task Force for Geriatric Emergency Medicine.(19) We developed 6 education sessions of 15 minutes each on the following topics: ‘Background of older patients visiting the ED’, ‘Vital signs in older patients’, ‘Cognitive disorders and delirium’, ‘Atypical presentations of older patients’, ‘How to administer the APOP-screener’ and ‘Interventions for high risk patients’. During one month before the kick-off of the APOP screening program all topics were presented several times to the ED nurses and physicians before every ED dayshift.

**Post-implementation phase**

After the kick-off of the APOP program at March 1^st^ 2018 we highlighted the APOP screening program at start of every dayshift in the ED to make personnel aware of screening. Every day one project-team member was available for questions. Screening rates, tips from the project-team and feedback from patients were displayed in the ED newsletter and information board in the ED. We also collected feedback from ED personnel on the program at the end of the first screening month during a 3-hour session with ED physicians and nurses. From April 2^nd^ the data collection period for evaluation was started. During this 2 months we did not organize any education or feedback sessions and only observed routine care without interference from our project-team. After the data collection period we send out questionnaires to all ED nurses and physicians and collected their feedback on the program. Simultaneously, some modifications were made in the EHR, resulting in a clearer overview of patients screened and not yet screened during their ED stay.

**Appendix 2.** Overview of the APOP screening program advices for interventions

| **Result screening** | **Advices triage-nurse** | **Advices treating ED-nurse** | | **Advices treating physician in ED** | |
| --- | --- | --- | --- | --- | --- |
| **1. Low risk** | Routine care | Routine care | | Routine care | |
| **2. High risk on functional decline and/or mortality** | • Inform treating nurse and physician about high risk result. | • Provide the patient with adequate nutrition and prevent dehydration as soon as possible.  • Try to put the patient on a bed instead of a gurney.  • Call family or caregiver if the patient arrived alone in the ED.  • Soon anticipate on the patients destination after the ED visit and prevent a long length of stay in the ED. | Discharge | • Provide the patient with adequate nutrition and prevent dehydration as soon as possible.  • Soon anticipate on the patients destination after the ED visit and prevent a long length of stay in the ED. | Discharge |
|  |  |  | • Call the patient within 24h for telephone follow-up. |  | • Screening results are automatically communicated to the GP in the discharge letter. |
|  |  |  | Admission |  | Admission |
|  |  |  | • Try to let family members accompany the patient during admission to the hospital ward. |  | • The geriatric consulting team will receive an automatic order to arrange geriatric assessment during admission. |
| **3. Signs of impaired cognition** | • Inform treating nurse and physician about high risk result. | • Provide the patient with adequate nutrition and prevent dehydration as soon as possible.  • Try to put the patient on a bed instead of a gurney.  • Call family or caregiver if the patient arrived alone in the ED.  • Start delirium preventive measures:  - Nurse the patient in a quiet room with normal daylight.  - Reduce the degree of stressfulness and noise of the ED environment: reduce sensory over-stimulation, keep the door closed and minimize the number of care providers.  - Take impaired cognition into account during history taking and instructional conversations.  • Soon anticipate on the patients destination after ED visit and prevent a long length of stay in the ED. | Discharge | • Provide the patient with adequate nutrition and prevent dehydration as soon as possible.  • Try to minimize the number of care providers.  • Consider whether the patient has a delirium.  • Take impaired cognition into account during history taking and instructional conversations.  • Soon anticipate on the patients destination after ED visit and prevent a long length of stay in the ED. | Discharge |
|  |  |  | • Call the patient within 24h for telephone follow-up. |  | • Screening results are automatically communicated to the GP in the discharge letter. |
|  |  |  | Admission |  | Admission |
|  |  |  | • Try to let family members accompany the patient during admission to the hospital ward. |  | • The geriatric consulting team will receive an automatic order to arrange geriatric assessment during admission |
| **4. High risk on both domains (2 + 3)** | Advises are the same as all mentioned above. | Advises are the same as all mentioned above. | | Advises are the same as all mentioned above. | |

**Standards for Reporting Implementation Studies: the StaRI checklist**

| **Checklist item** | | **Implementation strategy** | **Intervention** | **Page** |
| --- | --- | --- | --- | --- |
| **Title** | **1** | Identification as an implementation study, and description of the methodology in the title and/or keywords | | 1 |
| **Abstract** | **2** | Identification as an implementation study, including a description of the implementation strategy to be tested, the evidence-based intervention being implemented and defining the key implementation and health outcomes | | 2 |
| **Introduction** | **3** | Description of the problem, challenge or deficiency in healthcare or public health that the intervention being implemented aims to address | | 4 |
|  | **4** | The scientific background and rationale for the implementation strategy (including any underpinning theory/framework/model, how it is expected to achieve its effects and any pilot work) | The scientific background and rationale for the intervention being implemented (including evidence about its effectiveness and how it is expected to achieve its effects) | 4 |
| **Aims and objectives** | **5** | The aims of the study, differentiating between implementation objectives and any intervention objectives | | 4 |
| **Methods: description** | **6** | The design and key features of the evaluation (cross-referencing to any appropriate methodology reporting standards), and any changes to study protocol, with reasons | | 6 |
|  | **7** | The context in which the intervention was implemented (consider social, economic, policy, healthcare, organisational barriers and facilitators that might influence implementation elsewhere) | | 6 |
|  | **8** | The characteristics of the targeted ‘site(s)’ (eg, locations/personnel/ resources, etc) for implementation and any eligibility criteria | The population targeted by the intervention and any eligibility criteria | 6-8 |
|  | **9** | A description of the implementation strategy | A description of the intervention | 6-8, appendix1, appendix2 |
|  | **10** | Any subgroups recruited for additional research tasks, and/or nested studies are described | | - |
| **Methods: evaluation** | **11** | Defined prespecified primary and other outcome(s) of the implementation strategy, and how they were assessed. Document any predetermined targets | Defined prespecified primary and other outcome(s) of the intervention (if assessed), and how they were assessed. Document any predetermined targets | 8-10 |
|  | **12** | Process evaluation objectives and outcomes related to the mechanism(s) through which the strategy is expected to work | | 8-10 |
|  | **13** | Methods for resource use, costs, economic outcomes and analysis for the implementation strategy | Methods for resource use, costs, economic outcomes and analysis for the intervention | - |
|  | **14** | Rationale for sample sizes (including sample size calculations, budgetary constraints, practical considerations, data saturation, as appropriate) | | 10-11 |
|  | **15** | Methods of analysis (with reasons for that choice) | | 11 |
|  | **16** | Any a priori subgroup analyses (eg, between different sites in a multicentre study, different clinical or demographic populations), and subgroups recruited to specific nested research tasks | | - |
| **Results** | **17** | Proportion recruited and characteristics of the recipient population for the implementation strategy | Proportion recruited and characteristics (if appropriate) of the recipient population for the intervention | 12 |
|  | **18** | Primary and other outcome(s) of the implementation strategy | Primary and other outcome(s) of the intervention (if assessed) | 12-13 |
|  | **19** | Process data related to the implementation strategy mapped to the mechanism by which the strategy is expected to work | | 12-13 |
|  | **20** | Resource use, costs, economic outcomes and analysis for the implementation strategy | Resource use, costs, economic outcomes and analysis for the intervention | *-* |
|  | **21** | *Representativeness and outcomes of subgroups including those recruited to specific research tasks* | | *-* |
|  | **22** | Fidelity to implementation strategy as planned and adaptation to suit context and preferences | Fidelity to delivering the core components of intervention (where measured) | appendix1 |
|  | **23** | Contextual changes (if any) which may have affected outcomes | | appendix1 |
|  | **24** | All important harms or unintended effects in each group | | 13 |
| **Discussion** | **25** | Summary of findings, strengths and limitations, comparisons with other studies, conclusions and implications | | 14-16 |
|  | **26** | Discussion of policy, practice and/or research implications of the implementation strategy (specifically including scalability) | Discussion of policy, practice and/or research implications of the intervention (specifically including sustainability) | 15 |
| **General** | **27** | Include statement(s) on regulatory approvals (including, as appropriate, ethical approval, confidential use of routine data, governance approval), trial or study registration (availability of protocol), funding, and conflicts of interest | | 17 |
